# Clinical and Epidemiological Features and Antimicrobial Susceptibility Patterns of *Chryseobacterium* Species: A Scoping Review

**DOI:** 10.3390/medicina61071197

**Published:** 2025-06-30

**Authors:** Chienhsiu Huang

**Affiliations:** Department of Internal Medicine, Dalin Tzu Chi Hospital, Buddhist Tzu Chi Medical Foundation, No. 2, Min-Sheng Road, Dalin Town, Chiayi County 622401, Taiwan; hgssport@yahoo.com.tw or dm550671@tzuchi.com.tw; Tel.: +886-9-21552418

**Keywords:** *Chryseobacterium* species, *Chryseobacterium indololgenes*, *Chryseobacterium gleum*, antimicrobial susceptibility

## Abstract

*Background and Objectives:* Infections with *Chryseobacterium* species are rare, and the susceptibility patterns of these species to antimicrobial agents are unclear. Therefore, the aim of this study was to explore the clinical and epidemiological features and antimicrobial susceptibility patterns of *Chryseobacterium* species by reviewing previous research on the antibiograms of *Chryseobacterium* species and the illnesses caused by *Chryseobacterium* species. *Materials and Methods:* A comprehensive search of the PubMed and Web of Science databases was conducted for all studies that investigated antimicrobial susceptibility patterns of *Chryseobacterium* species published between January 1990 and February 2025. An extensive review of the infection incidences, isolation sites, clinical characteristics, and antimicrobial susceptibility patterns for infections caused by *Chryseobacterium* species was performed. *Results:* Several studies have revealed that the incidence of *Chryseobacterium* species infections is increasing, particularly in patients with comorbid conditions, mainly cardiovascular disease, diabetes mellitus, and malignancy. Most patients were elderly individuals, and most related illnesses were acquired in hospitals. The number of patients who received inappropriate antimicrobial therapy outnumbered the number of those who died. Antibiotics had little effect on *Chryseobacterium* species infection outcomes. Sixteen studies were included in the current scoping review. The susceptibility rates of *Chryseobacterium indologenes* to piperacillin/tazobactam (2.9–100%), ciprofloxacin (4.34–85%), levofloxacin (8.69–100%), trimethoprim/sulfamethoxazole (33.3–100%), imipenem (0–33.3%), meropenem (0–38.8%), minocycline (30.4–100%), ceftazidime (0–100%), and cefepime (0–100%) varied. The susceptibility rates of *Chryseobacterium gleum* to piperacillin/tazobactam (0–33%), ciprofloxacin (21.4–40%), levofloxacin (59.5%), trimethoprim/sulfamethoxazole (57.1–93.3%), imipenem (0–2.4%), meropenem (0%), minocycline (83.3–100%), ceftazidime (0–23.8%), and cefepime (0–19.0%) varied. *Conclusions:* Morbidity and mortality due to the increasing incidence of *Chryseobacterium* species infections have considerably increased. Underlying immunological defenses and other clinical factors may influence the prognosis of *Chryseobacterium* species infection. Rather than bacterial virulence characteristics, host factors mostly affect patient outcomes. Most isolates of *Chryseobacterium indologenes* are susceptible to minocycline and trimethoprim/sulfamethoxazole. For the treatment of these infections, professional knowledge and therapeutic expertise must be integrated.

## 1. Introduction

Previously classified as belonging to the genus *Flavobacterium*, *Chryseobacterium* species are a group of Gram-negative bacilli. These microorganisms are frequently associated with plant and animal diseases [1]. The genus *Chryseobacterium* has 182 species [2]. Previously referred to as *Flavobacterium* CDC group IIb, *F. indologenes*, and *F. gleum* [3]. Both soil and water environments are typical habitats for these species. Despite proper chlorination, *Chryseobacterium* species frequently colonize sink basins, taps, and water sources in the hospital environment, and *Chryseobacterium* species are commonly retrieved from damp surfaces and water sources [4]. These species were first identified as an etiology of opportunistic infection in 1993 in a patient suffering from ventilator-associated pneumonia [5]. The most frequent species linked to infections in humans is *C. indologenes*. Infections with *C. gleum* are reported far less frequently than infections with *C. indologenes* [6]. The first clinical instance of a 16-year-old boy with acute lymphoblastic leukemia who also had a bloodstream infection caused by *C. hominis* was documented by Won D et al. in 2019 [7]. Hospitalized patients with risk factors such as underlying illnesses (diabetes or cancer), extreme age (newborns or elderly individuals), immunocompromised conditions, the presence of indwelling devices, and long-term use of broad-spectrum antibiotics are more likely to contract infections with *Chryseobacterium* species. Numerous conditions, including pneumonia, bacteremia, cellulitis, urinary tract infections, ocular infections, surgical wound infections, meningitis, peritonitis, endocarditis, infections of the skin and soft tissues, and other catheter-related infections, are caused by *C. indologenes* [8,9,10,11,12,13,14,15,16,17,18,19,20]. *C. indologenes* are accurately identified utilizing traditional biochemistry-based phenotyping techniques and matrix-assisted laser desorption ionization–time of flight mass spectrometry (MALDI-TOF MS). In contrast, neither Vitek MS systems nor commercial biochemically based phenotyping identifications can correctly identify *C. gleum*. *Chryseobacterium gleum* is frequently mistakenly recognized as *C. indologenes* by these microbial identification techniques. The low prevalence of *C. gleum* in clinical samples may be attributed to the failure of the Vitek 2 system (bioMérieux, Marcy l’Etoile, France) to distinguish *C. gleum* from similar bacteria, and these systems are widely used in clinical microbiology laboratories worldwide. Overall, a Bruker Biotyper MALDI-TOF MS system (Bruker Daltonics GmbH, Bremen, Germany) or 16S rRNA gene sequencing is required for the precise identification of *Chryseobacterium* species [21,22,23]. It is difficult to choose an appropriate antibiotic for treating *Chryseobacterium* species infections since there is no treatment guideline in the literature. Class A b-lactamase and class B carbapenem-hydrolyzing b-lactamase molecules are produced, which lead to intrinsic carbapenem and cephalosporin resistance. However, piperacillin–tazobactam, ceftazidime, cefepime, minocycline, and trimethoprim–sulfamethoxazole are often effective [4,24,25]. The newer quinolones may be the most appropriate antimicrobial agents for treating infections caused by *C. indologenes* [4]. Since *Chryseobacterium* is a rare pathogen, its antimicrobial susceptibility pattern is unclear. Neither the Clinical Laboratory Standards Institute (CLSI) nor the European Committee on Antimicrobial Susceptibility Testing (EUCAST) has provided antimicrobial susceptibility testing criteria for the genus *Chryseobacterium*. Therefore, the aim of this study was to explore the clinical and epidemiological features, as well as antimicrobial susceptibility patterns, of *Chryseobacterium* species by reviewing previous research on the antibiograms of *Chryseobacterium* species and the diseases caused by *Chryseobacterium* species.

## 2. Materials and Methods

All of the studies, including those that examined the antimicrobial susceptibility patterns of *Chryseobacterium* species, were found through a thorough search of the PubMed and Web of Science databases for relevant literature published from 1 January 1990, to 28 February 2025. The phrases “*Chryseobacterium* or *Chryseobacterium indologenes* or *Chryseobacterium gleum*” and “infection” or “antimicrobial susceptibility” or “antimicrobial therapy” were used as search terms. Each study was scanned, and its eligibility was assessed. Following the removal of duplicates, the abstracts and titles of every publication that was retrieved were reviewed to find records that were eligible. The eligibility of all pertinent publications was assessed by reading the whole texts after excluding research that was deemed unnecessary. From the full-text publications, details about the author, the country, the study period, and the overall number of antimicrobial susceptibility patterns were retrieved. Prospective, retrospective, and randomized controlled trials were included in the current scoping review. Only studies in which the antimicrobial susceptibility patterns of *Chryseobacterium* species were investigated were included. Only articles written in English were included. Furthermore, studies with fewer than ten isolates were not included. The CLSI has not determined the minimum inhibitory concentration (MIC) cut-offs for *Chryseobacterium* species. There is a dearth of information in the literature concerning the susceptibility of *Chryseobacterium* species to antibiotics. Research on the susceptibility of *Chryseobacterium* species to antibiotics has been incorporated into the current review. An extensive review of the incidences of infection, sites where *Chryseobacterium* species were isolated, clinical characteristics, and mortality of patients with infections caused by *Chryseobacterium* species was performed.

This review was performed in accordance with the PRISMA (Preferred Reporting Items for Systematic Reviews and Meta-Analyses) guidelines [26]. The Systematic Review and Meta-Analysis was registered at the Prospero international prospective register of systematic reviews (registration: CRD420251027724).

## 3. Results

**Figure 1:**
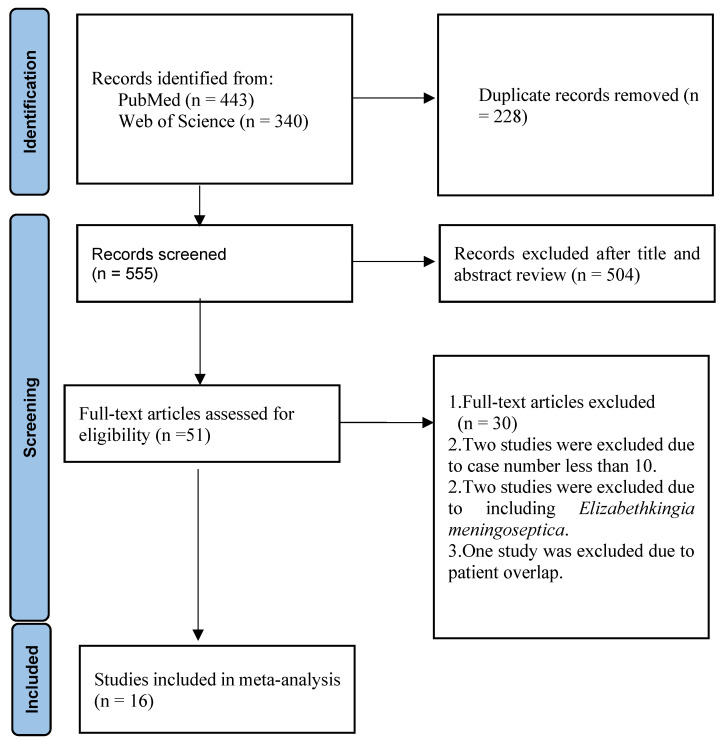
Flow diagram of the study selection process. Sixteen studies were included in the scoping review.

The specifics of the study selection procedure are shown in Figure 1. Fifty-one possibly relevant studies remained after duplicates and irrelevant research were eliminated. Following an examination of the full texts, 30 studies were excluded because of the absence of data concerning the antimicrobial susceptibility patterns of *Chryseobacterium* species. Two studies were excluded because the number of isolates was fewer than ten [27,28]. Two studies were excluded because they included *Elizabethkingia meningoseptica* [25,29]. Hsueh PR et al. conducted two investigations in the literature. The patients involved in both investigations overlapped. As a result, one study was excluded from the current meta-analysis [30]. Ultimately, 16 studies were included in the current scoping review [4,6,31,32,33,34,35,36,37,38,39,40,41,42,43,44]. Susceptibility was interpreted according to the MIC criteria for “other non-*Enterobacteriaceae*” and “*Pseudomonas aeruginosa*” in the CLSI guidelines by 13 studies [6,32,33,34,35,37,38,39,40,41,42,43,44]. Antimicrobial susceptibility was interpreted according to the MIC criteria in the National Committee for Clinical Laboratory Standards (NCCLS) by three studies [4,31,36]. Table 1 showed that the Risk of Bias in Non-randomized Studies of Interventions (ROBINS-I) tool was used to evaluate 16 studies.

Six studies were conducted to explore the antimicrobial susceptibility of *Chryseobacterium* species [6,31,32,33,34,35]. The susceptibility rates of *Chryseobacterium* species to piperacillin/tazobactam (5.27–100%), ciprofloxacin (0–94.4%), levofloxacin (0–94.4%), trimethoprim/sulfamethoxazole (33–100%), imipenem (0–33%), meropenem (0–33%), minocycline (73–100%), ceftazidime (0–100%), and cefepime (0–100%) varied (Table 2A,B).

Twelve studies were conducted to explore the antimicrobial susceptibility of *Chryseobacterium indologenes* [4,6,32,35,36,37,38,39,40,41,42,43]. The susceptibility rates of *Chryseobacterium* indologenes to piperacillin/tazobactam (2.9–100%), ciprofloxacin (4.34–85%), levofloxacin (8.69–100%), trimethoprim/sulfamethoxazole (33.3–100%), imipenem (0–33.3%), meropenem (0–38.8%), minocycline (30.4–100%), ceftazidime (0–100%), and cefepime (0–100%) varied (Table 3A,B).

Two studies were conducted to explore the antimicrobial susceptibility of *Chryseobacterium gleum* [6,44]. The susceptibility rates of *Chryseobacterium gleum* to piperacillin/tazobactam (0–33%), ciprofloxacin (21.4–40%), levofloxacin (59.5%), trimethoprim/sulfamethoxazole (57.1–93.3%), imipenem (0–2.4%), meropenem (0%), minocycline (83.3–100%), ceftazidime (0–23.8%), and cefepime (0–19.0%) varied (Table 4A,B).

However, antimicrobial susceptibility rates can be influenced by geographical location, antimicrobial susceptibility testing methods, and the time of bacterial isolation. We analyzed the relationship between antimicrobial susceptibility rates of *Chryseobacterium* species and geographical location, study period, and test method separately, as shown in Table 5, Table 6 and Table 7.

## 4. Discussion

### 4.1. Clinical and Epidemiological Features of Patients with Chryseobacterium Species Infections

#### 4.1.1. Incidence of Infection

Although several clinical cases have been reported in North America, South America, and Europe, an analysis of the literature revealed that the highest prevalence of *Chryseobacterium* species infections is in Asia. Lin JN et al. reported a notable increase in the incidence of *Chryseobacterium* infections between 2005 and 2017 [6]. An investigation by Chen FL et al. revealed that after 2006 [39], the incidence of *C. indologenes* infections progressively increased. There was a correlation between increasing consumption of colistin or tigecycline and the number of *C*. *indologenes* isolated [39]. Zhang Y et al.’s investigation revealed that from 2010 to 2016 [42], out of 135 clinical isolates, 39 were *C. indologenes*. Since then, the number of isolates has steadily increased. Sixty-six strains and thirty strains were collected in 2017 and 2018, respectively [42]. A rising incidence of *C. indologenes* bacteraemia over the past six years was documented in a study by Chou DW et al. (0 in 2003, 0.079 cases per 1000 patient-days in 2004, 0.155 in 2005, 0.154 in 2006, 0.154 in 2007, and 0.227 in 2008) [38]. Increased usage of broad-spectrum antibiotics such as tigecycline and colistin may lead to an increase in *C. indologenes* infections linked to healthcare. The current analysis revealed that there was a considerable increase in morbidity and mortality due to the increasing incidence of *Chryseobacterium* species infections in several studies. Significant issues have been caused by *Chryseobacterium* species in clinical healthcare settings.

#### 4.1.2. Sites of Isolation

Kirby JT et al. revealed that hospitalized patients were the source of two isolates of *C. gleum* and twenty isolates of *C. indologenes*. Fifteen isolates were from respiratory tract infections, and seven isolates were from bloodstream infections caused by *C. indologenes* [25]. Among the 126 isolates of *Chryseobacterium* species that were identified by Lin JN et al., 68 (54%) were collected from blood, 15 (11.9%) from bile, 10 (7.9%) from the tip of a central venous catheter, and 8 (6.3%) from urine [5]. According to the investigations of Chen FL et al., 215 *C. indologenes* isolates were found, including 138 from 91 patients’ sputum samples, 39 from 22 patients’ blood samples, and 38 from miscellaneous samples [39]. Zhang Y reported that the majority of 135 *C. indologenes* isolates were obtained from ascites (77/135, 57.0%), urine (32/135, 23.7%), sputum (18/135, 13.3%), bile (3/135, 2.2%), blood (2/135, 1.5%), and wound secretion samples (1/135, 0.7%), and 2 isolates for which information was missing were among the other isolates [42]. According to Kho MCY et al.’s research on bloodstream infections caused by the 25 identified *Chryseobacterium* species, 15 (60%) of the cases were associated with catheter-related bacteremia, 5 (20%) with intraabdominal infections, 4 (16%) with pneumonia, and 1 (4%) with skin and soft tissue infections [42]. In addition, urine was the most common source of samples according to four studies [35,40,41,44]. Blood was the most common sample source according to four studies [36,37,43]. Sputum was the second most common sample source according to three studies [40,42,44]. The current review revealed that the three most common sample sources of *Chryseobacterium* species isolates were (in order) blood, urine, and sputum.

#### 4.1.3. Clinical Characteristics of Patients with *Chryseobacterium* Species Infections

Male predominance (68.3%) was noted among the 126 patients with *Chryseobacterium* infections in the Lin JN et al. investigation. The median age was 59.5 years. The majority of patients (81.7%) had comorbid conditions, with heart disease being the most prevalent, followed by diabetes, malignancy, and liver cirrhosis. The majority of illnesses were related to healthcare (99.2%, 125/126) [5]. A male predominance (60.1%) was observed among the 113 patients with *C. indologenes* infections in Chen FL et al.’s investigation. The average age was greater than 70 years. Comorbidities were observed in most patients, with hypertensive cardiovascular disorders (61.9%) being the most common, followed by diabetes mellitus (32.7%), chronic kidney disease (32.7%), and malignancy (28.3%). All 113 individuals had healthcare-associated infections. *Acinetobacter baumannii* (36/91, 39.6%) was the most common coinfecting pathogen in patients with *C. indologenes* pneumonia, followed by *Pseudomonas aeruginosa* (23/91, 25.3%), carbapenem-resistant *Acinetobacter baumannii* (22/91, 24.2%), and *Klebsiella pneumoniae* (13/91, 14.3%) [39]. According to Zhang Y et al., the majority of patients were male (97/135, 71.9%), the mean age was 55 years (range: 5–98 years), and 36 (26.7%) of the patients were older than 65 years. The majority of cases were caused by nosocomial infections (84.4%, 114/135) [42]. According to Kho MCY et al.’s study on bloodstream infections caused by *Chryseobacterium* species, the majority of patients (18/25, 72%) were male, and the mean age was 61.9 years. Most cases were caused by nosocomial infection (76%, 19/25). The majority of patients had comorbid conditions, with the most prevalent being immunocompromised (12/25, 48%), followed by hypertension (12/25, 48%), diabetes mellitus (10/25, 40%), ischemic heart disease (8/25, 32%), and malignancy (28.3%). In 11 instances, coinfections were found (44%) [33]. Additionally, numerous investigations have revealed the presence of coinfecting pathogens in patients infected with *Chryseobacterium* species [31,32,36,37,38,44]. The present review revealed that individuals with comorbid conditions, mainly cardiovascular disease, diabetes mellitus, and malignancy, have infections caused by *Chryseobacterium* species. The majority of the patients were elderly individuals, and most of these illnesses were acquired in hospitals. Numerous individuals who had infections caused by *Chryseobacterium* species had coinfections with other pathogens.

#### 4.1.4. Mortality of Patients with *Chryseobacterium* Species Infections

Lin JN et al. reported that the total mortality rate for those infected with *Chryseobacterium* species was 19.8% (55/126). In 88.9% (112/126) of the patients, inappropriate antimicrobial treatments were identified [5]. According to Kho MCY et al., 52% (13/25) of patients with *Chryseobacterium* bacteremia received inappropriate antibiotic treatments, and the in-hospital mortality rate for these patients was 20% (5/25) [33]. According to Hsueh PR et al.’s study, 13.9% of those who had *C. indologenes* infections died (5/36). In 69.4% (25/36) of patients, inappropriate antimicrobial treatments were identified [36]. Lin YT et al. reported that 6.3% (1/16) of those with *C. indologenes* bacteraemia died. In 81.2% (3/16) of patients, inappropriate antibiotic treatments were identified [37]. According to Chou DW et al.’s study, 40% of those with *C. indologenes* bacteremia died (4/10). Eight out of ten patients (80%) had inappropriate antibiotic treatment [38]. According to Chen FL et al.’s study, 55.7% of patients received inappropriate antibiotic treatment. The overall in-hospital mortality rate of those with *C. indologenes* infections was 40.7% (46/113). Twenty patients received appropriate antibiotic therapy (mortality rate 40% = 20/50), and twenty-six patients received inappropriate antibiotic therapy (mortality rate 41.2% = 26/63) [39]. According to Deng L. et al., 17.4% of those who had *C. indologenes* infections died (4/23). In 78.2% (18/23) of patients, inappropriate antibiotic treatments were identified [41]. *Chryseobacterium* species infections were associated with a high mortality rate, with reported rates ranging from 6.3% to 40.7%, and inappropriate antibiotic treatment was identified in 52% to 88.9% of patients in the current review. The number of patients who received inappropriate antimicrobial therapy outnumbered the number of those who died. Antibiotics have little effect on the outcome of *Chryseobacterium* species infections. Underlying immunological defenses and other clinical factors may influence the prognosis of *Chryseobacterium* species infections. In addition to bacterial virulence characteristics, host factors mostly affect patient outcomes.

Hsueh PR et al. reported that four out of six patients with monomicrobial intravascular catheter-related bacteremia caused by *C. indologenes* experienced clinical improvement while the catheters were in place; these patients received only adequate antibiotic treatment. *Chryseobacterium indologenes* is a clinically benign condition that does not require catheter removal [36]. Whether indwelling catheters should be removed when there is *C. indologenes* infection is a matter of debate. There are conflicting reports on the efficacy of antibiotic therapy, both with and without the removal of the indwelling device [9,45,46,47]. In general, when adequate antimicrobial therapy fails, indwelling catheters should be withdrawn. The device does not need to be removed if infection with *C. indologenes* does not result in a rapid decline in clinical status [45,48]. However, removing the indwelling device may speed up recovery for certain immunocompromised individuals [49].

### 4.2. Antimicrobial Susceptibility Patterns

#### 4.2.1. Antimicrobial Susceptibility Patterns of *Chryseobacterium* Species

The findings of the thorough examination of the susceptibility of *Chryseobacterium* species to antibiotics differed depending on the region. Piperacillin–tazobactam, ciprofloxacin, levofloxacin, trimethoprim–sulfamethoxazole, ceftazidime, and cefepime all demonstrated high potency of action against *Chryseobacterium* species, according to the study by Mirza HC et al. The majority of the isolates in Mirza’s investigation were obtained from pediatric cystic fibrosis outpatients. The E test is a method for antimicrobial susceptibility testing. Antimicrobial resistance testing methods, pediatric patient groups, and region-specific antibiotic usage patterns can all contribute to high rates of antibiotic susceptibility [32]. As a result, the study by Mirza HC et al. is not included in the discussion below.

No research has demonstrated that the rate of antimicrobial susceptibility exceeds 40%, and the susceptibility to imipenem and meropenem is very low [6,31,32,33,34,35]. The use of imipenem and meropenem to treat patients with infections caused by *Chryseobacterium* species is not advised.

There were differences in the susceptibility to piperacillin–tazobactam. Four investigations reported that the susceptibility was less than 50% [6,31,34,35], whereas only one study reported that the susceptibility was 80% [33]. The use of piperacillin–tazobactam to treat patients with infections caused by *Chryseobacterium* species is not advised. The susceptibility to ciprofloxacin is fairly low, and no research has demonstrated that ciprofloxacin susceptibility is greater than 50% [6,31,32,33,34]. The susceptibility to levofloxacin varied. Three investigations revealed that the susceptibility to levofloxacin was less than 50% [6,31,34], whereas one study reported that the susceptibility was 77.3% [33]. The use of ciprofloxacin and levofloxacin to treat patients with infections caused by *Chryseobacterium* species is not advised. The susceptibility to ceftazidime and cefepime is very low, and no research has demonstrated that the antimicrobial susceptibility exceeds 30% [6,31,33,34,35]. The use of ceftazidime and cefepime to treat patients with infections caused by *Chryseobacterium* species is not advised.

There were differences in the susceptibility to trimethoprim–sulfamethoxazole. Two investigations revealed that the susceptibility to trimethoprim–sulfamethoxazole was less than 50% (cotrimoxazole was used in one study) [5,35], whereas three studies revealed that the susceptibility was greater than 70% (cotrimoxazole was used in one study) [31,33,34]. The pooled rate of susceptibility to trimethoprim/sulfamethoxazole was 59.5%. Treating patients with infections caused by *Chryseobacterium* species with trimethoprim–sulfamethoxazole is questionable. The susceptibility to minocycline was good. According to three investigations, the susceptibility exceeded 70% [6,33,34]. In the Lin JN et al. investigation, *Chryseobacterium* species isolates were shown to be the most susceptible to minocycline [6]. Minocycline may be considered an option for the treatment of urinary tract infections caused by *Chryseobacterium* species, according to the study by Kaur H et al. [34]. The pooled rate of susceptibility to minocycline was 79.0%. Minocycline may be suitable for the treatment of *Chryseobacterium* species infections based on in vitro susceptibility.

#### 4.2.2. Antimicrobial Susceptibility Patterns of *Chryseobacterium indologenes*

The findings of the thorough examination of the susceptibility of *C. indologenes* to antibiotics differed according to the region. Piperacillin–tazobactam, ciprofloxacin, levofloxacin, trimethoprim–sulfamethoxazole, ceftazidime, and cefepime all showed a high potency of action against *C. indologenes*; according to research by Kirby JT et al., only 20 isolates were identified. The results from more than 119 sentinel hospitals and laboratories in North America, Latin America, Europe, and the Asia-Pacific area during the first five years of the program (1997 to 2001) were used in this study. The early study period may have led to high rates of antibiotic susceptibility. Furthermore, 20 isolates cannot serve as a global reference standard [4]. Piperacillin–tazobactam, ciprofloxacin, levofloxacin, trimethoprim–sulfamethoxazole, ceftazidime, and cefepime all showed high potencies of action against *C. indologenes* according to research by Mirza HC et al. [32]. As a result, the studies by Kirby JT et al. and Mirza HC et al. are not included in the discussion below [4,32]. The susceptibility to imipenem and meropenem is very low, and no research has demonstrated that the antimicrobial susceptibility exceeds 40% [4,5,35,36,37,38,39,40,41,42,43]. It is not advised to treat patients with *C. indologenes* infections with imipenem and meropenem. The susceptibility to piperacillin–tazobactam is fairly low. Eight investigations revealed that the susceptibility is less than 50% [6,35,38,39,40,41,42,43], whereas only one study revealed that the susceptibility was 50% [37]. Therefore, treating patients with *C. indologenes* infections with piperacillin–tazobactam is not advised. The susceptibility to ciprofloxacin is fairly low, and no research has demonstrated that susceptibility to ciprofloxacin is greater than 50% [6,35,36,37,38,39,40,41,42,43]. Therefore, treating patients with *C. indologenes* infections with ciprofloxacin is not advised. The susceptibility to levofloxacin varied. Five other studies revealed that the susceptibility was less than 40% [6,38,39,41,42], whereas two investigations revealed that the susceptibilities were 62.5% and 75.0% [37,43]. The pooled rate of susceptibility to levofloxacin was 26.5%. Therefore, the use of levofloxacin to treat patients with *C. indologenes* infections is not advised. The susceptibility to ceftazidime and cefepime is relatively low, and no research has demonstrated that the antimicrobial susceptibility is greater than 50% [6,35,36,37,38,39,40,41,42,43]. Therefore, the use of ceftazidime and cefepime to treat patients with infections caused by *C. indologenes* is not advised. There were differences in the susceptibility to trimethoprim–sulfamethoxazole. Three investigations revealed that the susceptibility to trimethoprim–sulfamethoxazole was less than 60% (cotrimoxazole was used in one study) [6,35,40], whereas six studies revealed that the susceptibility was greater than 70% [37,38,39,41,42,43]. According to Lin YT et al., trimethoprim–sulfamethoxazole may be the most appropriate antibiotic for treating infections caused by *C. indologenes*. Chen FL et al. concluded that piperacillin–tazobactam and newer fluoroquinolones are no longer effective because of the decreased susceptibility of *C. indologenes* to these drugs. However, trimethoprim–sulfamethoxazole remains a reliable antimicrobial agent for treating *C. indologenes* infections [39]. Zhang Y et al. reported that the best antimicrobial drugs for treating infections caused by *C. indologenes* are trimethoprim–sulfamethoxazole and minocycline [42]. The pooled rate of susceptibility to trimethoprim/sulfamethoxazole was 78.0%. Therefore, trimethoprim–sulfamethoxazole is considered suitable for the treatment of *C. indologenes* infections based on in vitro susceptibility.

The susceptibility to minocycline was good. Four investigations revealed that the susceptibility to minocycline was greater than 70% [36,38,40,42], whereas two other studies revealed that the susceptibilities were 67.9% and 30.4% [6,41]. Chang YC reported that minocycline was the most effective antimicrobial agent against *C. indologenes* infections. Zhang Y et al. reported that minocycline and trimethoprim–sulfamethoxazole were the most suitable antimicrobial drugs for treating *C. indologenes* infections [42]. The pooled rate of susceptibility to minocycline was 83.2%. Therefore, minocycline is advised for the treatment of patients with *C. indologenes* infections based on in vitro susceptibility.

#### 4.2.3. Antimicrobial Susceptibility Patterns of *Chryseobacterium gleum*

The antimicrobial susceptibility patterns of *C. gleum* have been described in only two studies. According to one study, 93.3% of isolates were susceptible to the trimethoprim–sulfamethoxazole [44]. According to two studies [6,44], the susceptibilities to minocycline were 100% and 83.3%. The pooled rate of susceptibility to trimethoprim/sulfamethoxazole was 66.7%. The pooled rate of susceptibility to minocycline was 87.7% (Table 4B). Treating patients with infections caused by *C. gleum* with trimethoprim/sulfamethoxazole is questionable. Minocycline may be appropriate for treating patients with *C. gleum* infections based on in vitro susceptibility.

#### 4.2.4. Analysis of the Relationship Between Antimicrobial Susceptibility Rate and Geographical Location, Antimicrobial Susceptibility Test Method, and Study Period

Regarding the relationship between antimicrobial susceptibility rate and geographical location, piperacillin/tazobactam and levofloxacin demonstrated high rates of antibiotic susceptibility to *Chryseobacterium* species in Singapore (only one study) [33]. Ciprofloxacin and levofloxacin demonstrated low rates of antibiotic susceptibility to *Chryseobacterium* species in India (only one study) [34]. Levofloxacin demonstrated a high rate of antibiotic susceptibility to *Chryseobacterium indololgenes* in India (only one study) [43]. Trimethoprim/sulfamethoxazole demonstrated a low rate of antibiotic susceptibility to *Chryseobacterium indololgenes* in India, which was due to the low susceptibility rate (33.3%) in the study of Yadav VS et al. [35]. Due to the small number of included studies and isolates, these are only observational findings, and no inferences can be made.

Regarding the relationship between antimicrobial susceptibility rate and antimicrobial susceptibility testing methods, in the study by Koh MCY et al., piperacillin/tazobactam, levofloxacin, and trimethoprim/sulfamethoxazole showed high antibiotic susceptibility against *Chryseobacterium* spp. using the E-test [33]. In the study by Lin YT et al., levofloxacin showed high antibiotic susceptibility against *Chryseobacterium indololgenes* using disc diffusion [37]. Due to the small number of included studies and isolates, these are only observational findings, and no inferences can be made.

Regarding the relationship between antimicrobial susceptibility rate and study period, the studies before 2010 showed that ciprofloxacin demonstrated a 22.6% susceptibility rate to *Chryseobacterium* species. The studies after 2010 showed that ciprofloxacin demonstrated all resistant to *Chryseobacterium* species. However, it is not advised to treat patients with *Chryseobacterium* species with ciprofloxacin.

Antimicrobial susceptibility rates are certainly affected by geographical location, antimicrobial susceptibility testing methods, and the time of bacterial isolation. This review was only able to include 16 studies and is certainly unable to draw further conclusions.

## 5. Limitations

This review has several limitations. Initially, medical experts conducted randomized controlled trials, which is difficult given the rarity of *Chryseobacterium* species infections. Our analysis included sixteen studies, all of which were retrospective in nature. All of the studies had a high risk of bias. The second limitation of this review is the small number of *Chryseobacterium* species-related infection cases that were included. Only three studies included more than 50 isolates. The majority of research has been carried out in Asia. Owing to the small number of isolates and their geographic location, bias was present. Third, only articles written in English were included; while this is acceptable, it does introduce language bias. Fourth, if studies relied solely on the Vitek 2 system in clinical microbiology laboratories, I would not exclude these studies from the current review, which was another limitation. Fifth, in the study of Fraser SL et al., 58 clinical isolates were tested with 23 antibiotics using the broth microdilution procedure. The results of the broth microdilution were contrasted with those obtained for vancomycin and piperacillin–tazobactam by agar dilution, E-test, and disk diffusion. There were 7.1 and 17.9% very severe mistakes with piperacillin–tazobactam by agar dilution and E-test, respectively, in comparison to the broth microdilution results [25]. The results of testing the susceptibility of *Chryseobacterium* species to antibiotics using various methods vary. A more precise method for determining susceptibility is broth microdilution. The broth microdilution method was not used for testing in all of the included studies.

## 6. Conclusions

The incidence of *Chryseobacterium* species infections has increased. The majority of the patients were elderly individuals, and most of these illnesses were acquired in hospitals. Patient outcomes are mostly determined by host factors rather than bacterial virulence factors. The optimal antimicrobial drugs for the treatment of nosocomial *Chryseobacterium* species are difficult to determine. The majority of *C. indologenes* isolates were susceptible to minocycline and trimethoprim/sulfamethoxazole. Minocycline and trimethoprim–sulfamethoxazole seem to be appropriate antimicrobial therapies based on in vitro susceptibility.

## 7. Future Directions

Minocycline and trimethoprim–sulfamethoxazole were shown to be effective against *C. indologenes* infections. The effects of minocycline and trimethoprim–sulfamethoxazole on infections caused by *C. indologenes* have unfortunately not been investigated in a multicenter randomized controlled trial. For the treatment of these infections, professional knowledge and therapeutic expertise must be integrated.

## Figures and Tables

**Table 1:** Risk bias of sixteen studies.

Author /Period	Confounding	Selection	Interventions Classification	Interventions Deviations	Missing Data	Measurement of Outcomes	Selective Results
Kirby JT/1997–2001 [4]	low risk	high risk	moderate risk	moderate risk	high risk	low risk	moderate risk
Lin LN/2005–2017 [6]	low risk	low risk	moderate risk	moderate risk	low risk	low risk	low risk
Lambiase A/2002–2006 [31]	moderate risk	moderate risk	high risk	high risk	high risk	serious risk	high risk
Mirza HC/2012–2016 [32]	high risk	serious risk	high risk	high risk	serious risk	serious risk	high risk
Koh MCY/2012–2024 [33]	high risk	serious risk	high risk	high risk	serious risk	serious risk	high risk
Kaur H/2013–2013 [34]	serious risk	serious risk	serious risk	high risk	serious risk	serious risk	serious risk
Yadav VS/2017–2019 [35]	serious risk	serious risk	serious risk	serious risk	serious risk	serious risk	serious risk
Hsueh PR/1993–1995 [36]	moderate risk	moderate risk	high risk	high risk	high risk	serious risk	high risk
Lin YT/2002–2008 [37]	serious risk	high risk	serious risk	serious risk	serious risk	serious risk	serious risk
Chou DW/2004–2008 [38]	serious risk	Serious risk	high risk	serious	serious risk	serious risk	serious risk
Chen FL/2004–2011 [39]	low risk	low risk	high risk	moderate risk	low risk	moderate risk	moderate risk
Chang YC/2007–2011 [40]	high risk	moderate risk	moderate risk	moderate risk	moderate risk	moderate risk	moderate risk
Deng L/2010–2013 [41]	high risk	high risk	high risk	high risk	high risk	high risk	high risk
Zhang Y/2010–2018 [42]	low risk	low risk	low risk	moderate risk	low risk	low risk	low risk
Jain V/2016–2016 [43]	high risk	high risk	high risk	moderate risk	high risk	high risk	high risk
Lo HH/2007–2011 [44]	high risk	moderate risk	high risk	high risk	high risk	high risk	high risk

**Table 2:** (**A**) Antimicrobial susceptibility patterns of *Chryseobacterium* species. (**B**) Antimicrobial susceptibility patterns of *Chryseobacterium* species.

(**A**)										
**Author/Period**	**Country/No.**	**TZP**	**CIP**	**LVX**	**SXT**	**IMI**	**MPM**	**MIN**	**CAZ**	**FEM**
Lambiase A/2002–2006 [31] ^%^*	Italy/30	45.5%	45.5%	45.5%	100%	0%	4.6%	NA	0%	0%
Lin LN/2005–2017 [6] *	Taiwan/126	19.8%	18.3%	32.5%	47.6%	0%	0%	73.0%	13.5%	17.5%
Mirza HC/2012–2016 [32] ^&^	Turkey/18	100%	94.4%	94.4%	100%	16.6%	16.6%	NA	100%	100%
Koh MCY/2012–2024 [33] ^&^	Singapore/25	80%	NA	77.3%	95.7%	NA	NA	100%	25%	NA
Kaur H/2013–2013 [34] ^%^	India/19	5.27%	0%	0%	73.6% ^$^	0%	5.27%	100%	0%	0%
Yadav VS/ 2017–2019 [35] ^%^	India/20	45%	NA	NA	33% ^$^	33%	33%	NA	NA	NA
(**B**)										
**Author/Period**	**Country**	**TZP**	**CIP**	**LVX**	**SXT**	**IMI**	**MPM**	**MIN**	**CAZ**	**FEM**
Lambiase A/2002–2006 [31] ^%^*	Italy	10/22	10/22	10/22	22/22	0/22	1/22	NA	0/22	0/22
Lin LN/2005–2017 [5] *	Taiwan	25/126	23/126	41/126	60/126	0/126	0/126	92/126	17/126	22/126
Koh MCY/2012–2024 [33] ^&^	Singapore	12/15	NA	17/22	22/23	NA	NA	17/17	1/4	NA
Kaur H/2013–2013 [34] ^%^	India	1/19	0/19	0/19	14 ^$^/19	0/19	1/19	19/19	0/19	0/19
Yadav VS /2017–2019 [35] ^%^	India	9/20	NA	NA	7 ^$^/20	7/20	7/20	NA	NA	NA
pooled rate		57/202	33/167	68/189	125/210	7/187	9/187	128/162	18/171	22/167
percentage		28.2%	19.8%	36.0%	59.5%	3.7%	4.8%	79.0%	10.5%	13.2%

Foot notes: TZP (piperacillin/tazobactam), CIP (ciprofloxacin), LVX (levofloxacin), MIN (minocycline), SXT (trimethoprim/sulfamethoxazole), IMI (imipenem), MPM (meropenem), CAZ (ceftazidime), FEM (cefepime), No. (number), NA (not applicable). *: Broth microdilution was used to conduct antimicrobial susceptibility testing. ^%^: Disc diffusion was used to conduct antimicrobial susceptibility testing. ^&^: E-test was used to conduct antimicrobial susceptibility testing. ^$^: Cotrimoxazole.

**Table 3:** (**A**) Antimicrobial susceptibility patterns of *Chryseobacterium indololgenes*. (**B**) Antimicrobial susceptibility patterns of *Chryseobacterium indololgenes*.

(**A**)										
**Author/Period**	**Country/No.**	**TZP**	**CIP**	**LVX**	**SXT**	**IMI**	**MPM**	**MIN**	**CAZ**	**FEM**
Kirby JT/1997–2001 [4] *	World/20	90%	85%	100%	95%	15%	10%	NA	85%	85%
Hsueh PR/1993–1995 [36] ^#^	Taiwan/36	NA	18%	NA	NA	0%	NA	74%	42%	NA
Lin YT/2002–2008 [37] ^%^	Taiwan/16	50%	43.7%	62.5%	75%	0%	0%	NA	0%	12.5%
Chou DW/2004–2008 [38] *	Taiwan/10	20%	30%	30%	100%	10%	0%	100%	0%	0%
Chen FL/2004–2011 [39] *^%^	Taiwan/177	29.3%	31.6%	34.4%	87.5%	3.9%	8.4%	NA	3.3%	3.3%
Lin JN/2005–2017 [6] *	Taiwan/84	13.1%	16.7%	19.0%	42.9%	0%	0%	67.9%	8.3%	16.7%
Chang YC/2007–2011 [40] *	China/34	2.9%	14.7%	NA	52.9%	0%	NA	100%	2.9%	2.9%
Deng L/2010–2013 [41] *^%^	China/23	26.0%	4.34%	8.69%	73.9%	8.69%	NA	30.4%	NA	13.0%
Zhang Y/2010–2018 [42] *	China/135	37.0%	12.6%	14.8%	97.8%	0.7%	0%	98.5%	6.7%	NA
Mirza HC/2012–2016 [32] ^&^	Turkey/16	100%	100%	100%	100%	18.8%	18.8%	NA	100%	100%
Jain V/2016–2016 [43] *	India/12	16.7%	41.6%	75.0%	91.6%	NA%	0%	NA	NA	0%
Yadav VS /2017–2019 [35] ^%^	India/18	44.4%	16.6%	NA	33.3% ^$^	33.3%	38.8%	NA	16.6%	NA
(**B**)										
**Author/** **Period**	**Country**	**TZP**	**CIP**	**LVX**	**SXT**	**IMI**	**MPM**	**MIN**	**CAZ**	**FEM**
Hsueh PR/1993–1995 [36] ^#^	Taiwan	NA	6/36	NA	NA	0/36	NA	27/36	15/36	NA
Lin YT/2002–2008 [37] ^%^	Taiwan	8/16	7/16	10/16	12/16	0/16	0/16	NA	0/16	2/16
Chou DW/2004–2008 [38] *	Taiwan	2/10	3/10	3/10	10/10	1/10	0/10	10/10	0/10	0/10
Chen FL/2004–2011 [39] *^%^	Taiwan	52/177	56/177	61/177	155/177	7/177	15/177	NA	6/177	6/177
Lin JN/2005–2017 [5] *	Taiwan	11/84	14/84	16/84	36/84	0/84	0/84	57/84	7/84	14/84
Chang YC/2007–2011 [40] *	China	10/34	5/34	NA	18/34	0/34	NA	34/34	1/34	1/34
Deng L/2010–2013 [41] *^%^	China	6/23	1/23	2/23	17/23	2/23	NA	7/23	NA	3/23
Zhang Y/2010–2018 [42] *	China	50/135	17/135	20/135	132/135	1/135	0/135	133/135	9/135	NA
Jain V/2016–2016 [43] *	India	2/12	5/12	9/12	11/12	NA	0/12	NA	NA	0/12
Yadav VS /2017–2019 [35] ^%^	India	8/18	3/18	NA	6 ^$^/18	6/18	7/18	NA	3/18	NA
pooled rate		149/509	117/545	121/457	397/509	17/533	22/452	268/322	41/510	26/356
percentage		29.3%	21.5%	26.5%	78.0%	3.2%	4.9%	83.2%	8.09%	7.3%

Foot notes: TZP (piperacillin/tazobactam), CIP (ciprofloxacin), LVX (levofloxacin), MIN (minocycline), SXT (trimethoprim/sulfamethoxazole), IMI (imipenem), MPM (meropenem), CAZ (ceftazidime), FEM (cefepime), No. (number), NA (not applicable). *: Broth microdilution was used to conduct antimicrobial susceptibility testing. ^#^: Agar dilution was used to conduct antimicrobial susceptibility testing. ^%^: Disc diffusion was used to conduct antimicrobial susceptibility testing. ^&^: E-test was used to conduct antimicrobial susceptibility testing. ^$^: Cotrimoxazole.

**Table 4:** (**A**) Antimicrobial susceptibility patterns of *Chryseobacterium gleum*. (**B**) Antimicrobial susceptibility patterns of *Chryseobacterium gleum*.

(**A**)										
**Author/Period**	**Country/No.**	**TZP**	**CIP**	**LVX**	**SXT**	**IMI**	**MPM**	**MIN**	**CAZ**	**FEM**
Lin JN/2005–2017 [6] *	Taiwan/42	33.3%	21.4%	59.5%	57.1%	2.4%	0%	83.3%	23.8%	19.0%
Lo HH/2007–2011 [44] *	Taiwan/15	0%	40.0%	NA	93.3%	0%	NA	100%	0%	0%
(**B**)										
**Author/Period**	**Country**	**TZP**	**CIP**	**LVX**	**SXT**	**IMI**	**MPM**	**MIN**	**CAZ**	**FEM**
Lin JN/2005–2017 [5] *	Taiwan	14/42	9/42	25/42	24/42	1/42	0/42	35/42	10/42	8/42
Lo HH/2007–2011 [44] *	Taiwan	0/15	6/15	NA	14/15	0/15	NA	15/15	0/15	0/15
pooled rate		14/57	15/57	25/42	38/57	1/57	0/42	50/57	10/57	8/57
percentage		24.6%	26.3%	59.5%	66.7%	1.8%	0%	87.7%	17.5%	14.0%

Foot notes: TZP (piperacillin/tazobactam), CIP (ciprofloxacin), LVX (levofloxacin), MIN (minocycline), SXT (trimethoprim/sulfamethoxazole), IMI (imipenem), MPM (meropenem), CAZ (ceftazidime), FEM (cefepime), No. (number), NA (not applicable). *: Broth microdilution was used to conduct antimicrobial susceptibility testing.

**Table 5:** (**A**) The relationship between *Chryseobacterium species* antimicrobial susceptibility rate and geographical location. (**B**) The relationship between *Chryseobacterium indololgenes* antimicrobial susceptibility rate and geographical location.

(**A**)									
**Country**	**TZP**	**CIP**	**LVX**	**SXT**	**IMI**	**MPM**	**MIN**	**CAZ**	**FEM**
Italy [31]	45.5%	45.5%	45.5%	100%	0%	4.6%	NA	0%	0%
Taiwan [5]	19.8%	18.3%	32.5%	47.6%	0%	0%	73.0%	13.5%	17.5%
Singapore [33]	80%	NA	77.3%	95.7%	NA	NA	100%	25%	NA
India [34,35]	25.6%	0%	0%	53.8%	17.9%	20.5%	100%	0%	0%
(**B**)									
**Country/No**	**TZP**	**CIP**	**LVX**	**SXT**	**IMI**	**MPM**	**MIN**	**CAZ**	**FEM**
Taiwan [6,36,37,38,39]	25.4%	26.6%	31.4%	74.2%	2.5%	5.2%	72.3%	8.7%	7.7%
China [40,41,42]	34.4%	12.0%	13.9%	87.0%	1.6%	0%	90.6%	5.9%	7.0%
India [35,43]	33.3%	26.7%	75.0%	56.7%	33.3%	23.3%	NA	16.7%	0%

Foot notes: TZP (piperacillin/tazobactam), CIP (ciprofloxacin), LVX (levofloxacin), MIN (minocycline), SXT (trimethoprim/sulfamethoxazole), IMI (imipenem), MPM (meropenem), CAZ (ceftazidime), FEM (cefepime), No. (number), NA (not applicable).

**Table 6:** (**A**) The relationship between *Chryseobacterium* species antimicrobial susceptibility rate and antimicrobial susceptibility testing methods. (**B**) The relationship between *Chryseobacterium indololgenes* antimicrobial susceptibility rate and antimicrobial susceptibility testing methods.

(**A**)									
**Method**	**TZP**	**CIP**	**LVX**	**SXT**	**IMI**	**MPM**	**MIN**	**CAZ**	**FEM**
E-test [33]	80.0%	NA	77.3%	95.7%	NA	NA	100%	25.0%	NA
Disc diffusion [34,35]	25.6%	0%	0%	53.8%	17.9%	20.5%	100%	0%	0%
Broth microdilution [5]	19.8%	18.3%	32.5%	47.6%	0%	0%	73.0%	13.5%	17.5%
(**B**)									
**Country**	**TZP**	**CIP**	**LVX**	**SXT**	**IMI**	**MPM**	**MIN**	**CAZ**	**FEM**
Agar dilution [36]	NA	16.7%	NA	NA	0%	NA	75.0%	41.7%	NA
Disc diffusion [35,37]	47.1%	29.4%	62.5%	52.9%	17.6%	20.6%	NA	8.8%	12.5%
Broth microdilution [5,38,40,42,43]	27.3%	16.0%	19.9%	75.3%	0.8%	0%	89.0%	6.5%	10.7%

Foot notes: TZP (piperacillin/tazobactam), CIP (ciprofloxacin), LVX (levofloxacin), MIN (minocycline), SXT (trimethoprim/sulfamethoxazole), IMI (imipenem), MPM (meropenem), CAZ (ceftazidime), FEM (cefepime), NA (not applicable).

**Table 7:** (**A**) The relationship between *Chryseobacterium* species antimicrobial susceptibility rate and study period. (**B**) The relationship between *Chryseobacterium indololgenes* antimicrobial susceptibility rate and study period.

(**A**)									
**Period**	**TZP**	**CIP**	**LVX**	**SXT**	**IMI**	**MPM**	**MIN**	**CAZ**	**FEM**
before 2010 [5,31]	24.0%	22.6%	34.9%	56.2%	0%	0.7%	73.0%	11.6%	15.1%
After 2010 [33,34,35]	40.7%	0%	41.5%	69.4%	17.9%	20.5%	100%	4.3%	0%
(**B**)									
**Period**	**TZP**	**CIP**	**LVX**	**SXT**	**IMI**	**MPM**	**MIN**	**CAZ**	**FEM**
Before 2000 [36]	NA	16.7%	NA	NA	0%	NA	75.0%	41.7%	NA
2000-2010 [5,37,38,39,40]	25.9%	26.5%	31.4%	72.0%	2.5%	5.2%	78.9%	4.4%	7.2%
After 2010 [35,41,42,43]	35.1%	13.8%	18.2%	88.3%	5.1%	4.2%	88.6%	7.8%	8.6%

Foot notes: TZP (piperacillin/tazobactam), CIP (ciprofloxacin), LVX (levofloxacin), MIN (minocycline), SXT (trimethoprim/sulfamethoxazole), IMI (imipenem), MPM (meropenem), CAZ (ceftazidime), FEM (cefepime), NA (not applicable).

## Data Availability

The datasets generated during and/or analyzed during the current study are not publicly available but are available from the corresponding author on reasonable request.
